# Large-scale quantum-emitter arrays in atomically thin semiconductors

**DOI:** 10.1038/ncomms15093

**Published:** 2017-05-22

**Authors:** Carmen Palacios-Berraquero, Dhiren M. Kara, Alejandro R.-P. Montblanch, Matteo Barbone, Pawel Latawiec, Duhee Yoon, Anna K. Ott, Marko Loncar, Andrea C. Ferrari, Mete Atatüre

**Affiliations:** 1 Cavendish Laboratory, University of Cambridge, JJ Thomson Avenue, Cambridge CB3 0HE, UK; 2 Cambridge Graphene Centre, University of Cambridge, Cambridge CB3 0FA, UK; 3 John A. Paulson School of Engineering and Applied Science, Harvard University, 29 Oxford Street, Cambridge, Massachusetts 02138, USA

## Abstract

Quantum light emitters have been observed in atomically thin layers of transition metal dichalcogenides. However, they are found at random locations within the host material and usually in low densities, hindering experiments aiming to investigate this new class of emitters. Here, we create deterministic arrays of hundreds of quantum emitters in tungsten diselenide and tungsten disulphide monolayers, emitting across a range of wavelengths in the visible spectrum (610–680 nm and 740–820 nm), with a greater spectral stability than their randomly occurring counterparts. This is achieved by depositing monolayers onto silica substrates nanopatterned with arrays of 150-nm-diameter pillars ranging from 60 to 190 nm in height. The nanopillars create localized deformations in the material resulting in the quantum confinement of excitons. Our method may enable the placement of emitters in photonic structures such as optical waveguides in a scalable way, where precise and accurate positioning is paramount.

## Abstract (web summary)

Quantum emitters have been recently isolated in 2D materials, yet their spatial controllability remains an open challenge. Here, the authors devise a method to create arrays of quantum emitters in WSe_2_ and WS_2_, by taking advantage of the strain distribution induced by a nanopatterned silica substrate.

Transition metal dichalcogenides (TMDs) are optically active, semiconducting layered materials (LMs) which can be exfoliated down to monolayers. These are of particular interest, as they exhibit properties such as an optically accessible valley degree of freedom that is locked to the exciton spin^1^,^2^,^3^, strong two-dimensional confinement, favouring bound excitonic states^4^,^5^ and the opportunity to investigate many-body physics^6^. Intrinsic properties of LMs—atomically precise interfaces, lack of dangling bonds, flexibility and the possibility of stacking different LMs into functional heterostructures^7^,^8^—make them not only interesting for fundamental physics but also suitable for technological applications^9^,^10^. For these reasons, the identification of quantum emitters (QEs) in LMs^11^,^12^,^13^,^14^,^15^,^16^ has generated much excitement in the field of two-dimensional nanophotonics^10^,^17^,^18^,^19^ and quantum technologies^9^,^20^.

Single photon emission has been seen from QEs in tungsten diselenide (WSe_2_) with both above bandgap^11^,^12^,^13^,^14^,^15^,^16^ and resonant optical excitation^21^. In addition, QEs in WSe_2_ and tungsten disulphide (WS_2_) have been implemented in heterostructures to achieve electrically driven single-photon emission^22^. However, the origin of QEs in TMDs is still unclear, and has been assigned to both defects^11^,^12^,^13^,^14^,^15^,^16^ and strain gradients^21^,^23^,^24^. Experiments on these QEs have, until now, been reliant on their rare and random occurrence. Deterministic creation of precisely positioned LM QEs in large numbers is important for accelerating the study of these emitters, as well as opening up the prospect for scalability and on-chip applications.

Here, we report a method to create arrays of single-photon emitting QEs in WSe_2_ and quantum-like emitters in WS_2_ using a nanopatterned silica substrate. We obtain structures with QE numbers typically in the range of hundreds. The quality of these deterministic QEs surpasses that of their randomly appearing counterparts, with spectral wanderings of ∼0.1 meV – an order of magnitude lower than previous reports^11^,^12^,^13^,^14^,^15^. Our technique is a crucial first step towards solving the scalability challenge for LM-based quantum photonic devices.

## Results

### Nanopatterned substrate preparation and characterization

**Figure 1:**
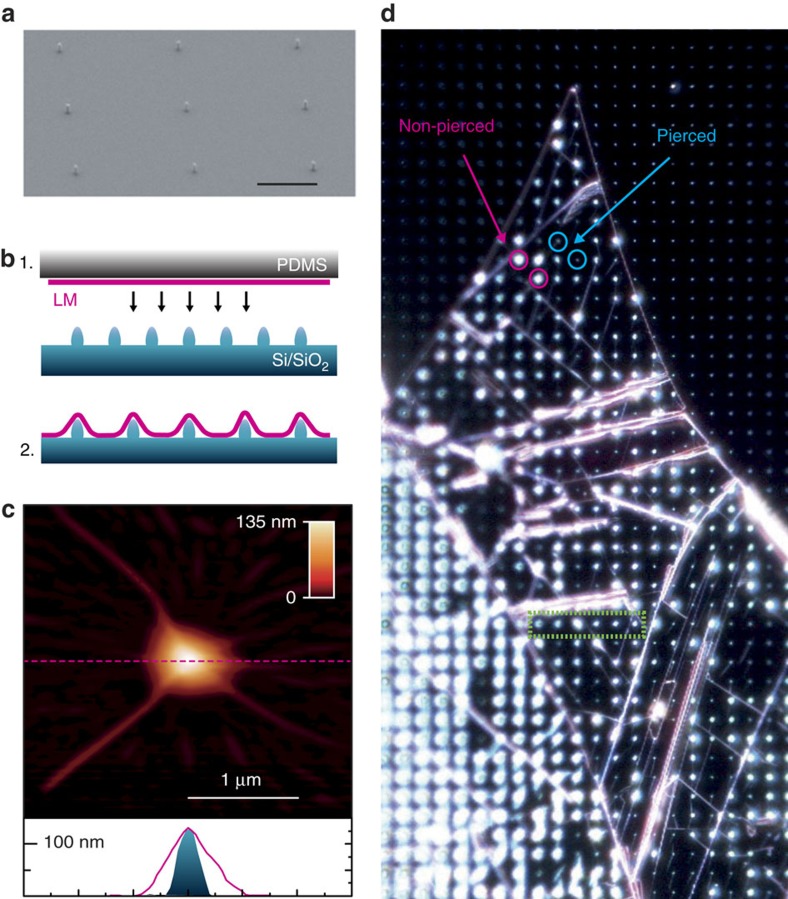
Fabrication and characterization of scalable quantum confinement arrays. (**a**) SEM image of nanopillar substrate, fabricated by electron beam lithography. Black scale bar, 2 μm. (**b**) Illustration of the fabrication method: (1) mechanical exfoliation of LM on PDMS and all-dry viscoelastic deposition on patterned substrate; and (2) deposited LM on patterned substrate. (**c**, top) An AFM scan of 1L-WSe_2_ on a nanopillar. (bottom) The AFM height profile of a bare nanopillar (blue-shaded region) and of the flake deposited over it (pink line), measured along the dashed pink line cut in the top panel. Colour-scale bar represents height in nm and white scale bar 1 μm. (**d**) Dark field optical microscopy image (real colour) of 1L-WSe_2_ on nanopillar substrate (130 nm high, 4 μm separation). The full image corresponds to a 170 μm by 210 μm area. The green box highlights six adjacent nanopillars within the 1L-WSe_2_ region, measured in Fig. 2. The blue circles indicate two pierced nanopillars, and the pink circles indicate two non-pierced nanopillars. PDMS, polydimethylsiloxane; SEM, scanning electron microscope.

To create large-scale QE arrays in LMs, we place the active material on patterned structures fabricated on the substrate in order to create spatially localized physical disturbances to the otherwise flat LM flakes. To this end, we first pattern arrays of nanopillars of different heights, ranging from 60 to 190 nm, on silica substrates using electron beam lithography. Figure 1a shows a scanning electron microscope image of one such substrate of 130 nm nanopillar height. We place layers of WSe_2_ and WS_2_ on the nanopillars as follows. Bulk WSe_2_ and WS_2_ crystals are characterized before exfoliation as described in ref. 22. These are then exfoliated on a polydimethylsiloxane layer by micromechanical cleavage^7^,^25^. Single-layer (1L) samples are identified first by optical contrast^26^, and the selected 1L-WSe_2_ and 1L-WS_2_ flakes are then placed onto the patterned nanopillar substrate via an all-dry viscoelastic transfer technique due to their higher adhesion to SiO_2_ (refs 7, 19, 27), as schematically shown in Fig. 1b. After exfoliation and transfer, the 1L-WSe_2_ and 1L-WS_2_ flakes are characterized by Raman spectroscopy^28^,^29^, photoluminescence (PL)^30^ and atomic force microscopy (AFM), confirming the transfer and that the process does not damage the samples (see Supplementary Fig. 1 and Supplementary Note 1 for the corresponding spectra and discussion). Figure 1c is an AFM scan of a 1L-WSe_2_ flake over a single nanopillar. The bottom panel of Fig. 1c plots the height profile of the 1L-WSe_2_ flake taken along the dashed pink line. This reveals how the flake (solid pink line) tents over the nanopillar. The blue-shaded area corresponds to the measured profile of a bare nanopillar. Figure 1d is a dark-field optical microscopy image of part of a 43,000 μm^2^ 1L-WSe_2_ flake on a substrate patterned with a 4-μm-spaced nanopillar array with nominal height of 130 nm. The regularly spaced bright spots correspond to nanopillar sites. We see locations providing brighter scattering (two examples are encircled in pink) and others showing fainter intensity (two examples are encircled in blue). By correlating with AFM measurements we find that the former correspond to locations where the 1L-WSe_2_ tents over the nanopillars and the latter correspond to locations where the flake is pierced by the nanopillars (see Supplementary Fig. 2). On average, we find that two-third of the sites are not pierced during the deposition step.

### Quantum light from WSe_2_-based deterministic QEs

**Figure 2:**
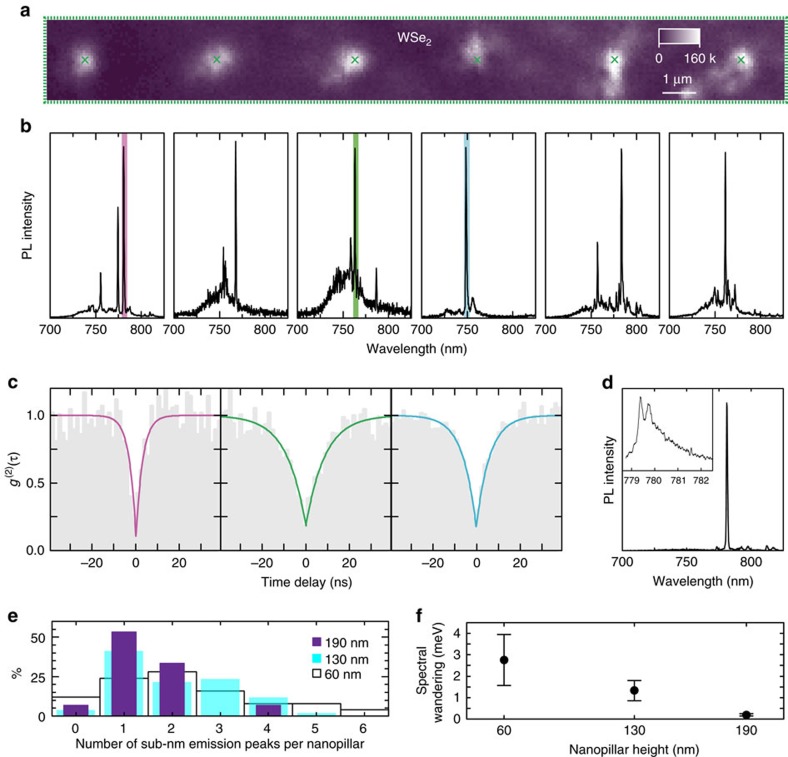
Creation of quantum emitter arrays in 1L-WSe_2_. (**a**) Integrated PL intensity raster scan of the region enclosed by the green rectangle in Fig. 1d, taken under 200 nW μm^−2^, 532 nm laser excitation at 10 K. Green crosses mark the position of the six nanopillars beneath the 1L-WSe_2_. Colour-scale bar maximum, 160 kcounts s^−1^. (**b**) PL spectra taken at each of the corresponding green crosses in **a**, from left to right respectively, showing the presence of narrow lines at each nanopillar location. (**c**) Photon correlation measurements corresponding to the filtered spectral regions (10 nm wide) enclosed by the blue, green and pink rectangles, in **b**, with *g*^(2)^(0)=0.087±0.065, 0.17±0.02 and 0.18±0.03, and rise times of 8.81±0.80 ns, 6.15±0.36 ns and 3.08±0.41 ns, respectively. (**d**) Spectrum taken from a 1L-WSe_2_ on a 190 nm nanopillar, showing lower background and a single sub-nm emission peak. Higher-resolution spectrum in the inset reveals the fine-structure splitting of this QE peak. An asymmetry can be seen in the spectrum, which has been previously attributed to a phonon sideband in naturally occurring QEs^31^. (**e**) Probability distribution (in %) of the number of emission lines per nanopillar for samples using different nanopillar heights (60, 130 and 190 nm in white, blue and purple, respectively). A trend of higher probability of single QE emission peaks per nanopillar location with increasing height is evident, reaching 50% for 190 nm nanopillars. (**f**) Increasing nanopillar height also leads to a reduction of spectral wandering. Solid black circles represent the mean value of spectral wandering of several QEs for a given nanopillar height, while the error bars represent the standard deviation of each distribution, both extracted from time-resolved high-resolution spectral measurements (Supplementary Fig. 4). A total number of seven samples was used to collect the statistics necessary for Fig. 2e,f.

Figure 2a is an integrated raster scan map of PL emission at ∼10 K of six adjacent non-pierced nanopillar sites in the region enclosed by the green dashed line in Fig. 1d. The most prominent feature is the ∼ × 10 increase in intensity at the location of every nanopillar. Figure 2b reveals the source of this emission intensity enhancement: spectra taken at each nanopillar location display bright sub-nanometre linewidth emission peaks. Figure 2c demonstrates the single-photon nature of this emission via photon-correlation measurements taken (from left to right) at the first, third and fourth nanopillar locations. Ten nanometre band-pass filters, indicated by the pink, green and blue highlighted areas in the panels of Fig. 2b, select the spectral windows for the photon-correlation measurements. We obtain *g*^(2)^(0) values of 0.0868±0.0645, 0.170±0.021 and 0.182±0.028, respectively, uncorrected for background emission or detector response. While these surpass those in early reports^11^,^12^,^13^,^14^,^15^, we expect the quality of the single-photon emission from the QEs to improve under resonant excitation^31^. Out of the 53 unpierced nanopillar sites in this substrate we find sub-nm emission peaks in 51 of them, giving ∼96% yield in QE generation. Their emission wavelength ranges between 730 and 820 nm (see Supplementary Fig. 3 for statistics), equivalent to a redshift distributed between 50 and 280 meV from the unbound exciton emission energy at ∼1.755 eV (ref. 32), as observed for the naturally occurring QEs in WSe_2_ (refs 11, 12, 13, 14, 15). The fine-structure splitting (200–730 μeV) and the emission linewidths as narrow as ∼180 μeV (∼0.08 nm) are also consistent with previous reports^11^,^12^,^13^,^14^,^15^ (Supplementary Fig. 3) advocating that these deterministically created QEs are of the same nature as the randomly appearing ones.

### Effect of nanopillar height on WSe_2_-based QEs

To study the effect of nanopillar height, we carry out similar optical measurements of 1L-WSe_2_ flakes deposited on nanopillars of height ∼60 and ∼190 nm. The spectra taken at the 60-nm nanopillars have multiple peaks of ∼1 nm linewidth on average (see Supplementary Fig. 3 for example spectra). In contrast, Fig. 2d is a representative spectrum taken from the 190 nm nanopillars, displaying a better isolated, single sub-nm emission peak. The inset reveals a 722 μeV fine-structure splitting for this QE. We do not see clear nanopillar height dependence in the emission wavelength and fine-structure splitting (see Supplementary Fig. 3 for statistics). Although we often observe multiple narrow emission lines from nanopillar locations, increasing the nanopillar height reduces the spread in the number of peaks arising at each. We verify this trend in Fig. 2e, a histogram of the probability that a given number of sub-nm emission peaks appear per nanopillar, for the different nanopillar heights (60, 130 and 190 nm in white, blue and purple, respectively). The likelihood of creating a single QE grows as nanopillar height is increased. For the 190 nm nanopillars, 50% of all nanopillar sites host a single QE with one emission peak, as indicated by the purple bars. Spectral wandering of the peaks as a function of time also displays a strong dependence on the nanopillar height. To quantify this dependence, we record the maximum range of emission wavelength wandering per QE over tens of seconds. The solid black circles in Fig. 2f correspond to the mean of these values for each group of QEs pertaining to each nanopillar height, for 17 different QEs in total, with the error bars displaying the standard deviation of these distributions. We observe a reduction from a few meV for 60 nm nanopillars to below 0.25 meV (average) for the tallest 190 nm nanopillars (see Supplementary Fig. 4), reaching as low as 0.1 meV. To the best of our knowledge, this is the lowest spectral wandering seen to date in LM QEs^11^,^12^,^13^,^14^,^15^. Hence, these deterministic QEs are comparable, and even superior, in spectral stability to their randomly appearing counterparts. The dependence of certain QE characteristics on nanopillar height, along with shifts in the delocalized neutral exciton peak (X^0^) at room temperature^30^ at the nanopillar locations (see Supplementary Fig. 5), suggest that a localized strain gradient induced by the nanopillars might be playing an active role in producing QEs, as well as determining their specific optical properties^21^,^23^,^24^.

### Deterministic QE creation in WS_2_ monolayers

**Figure 3:**
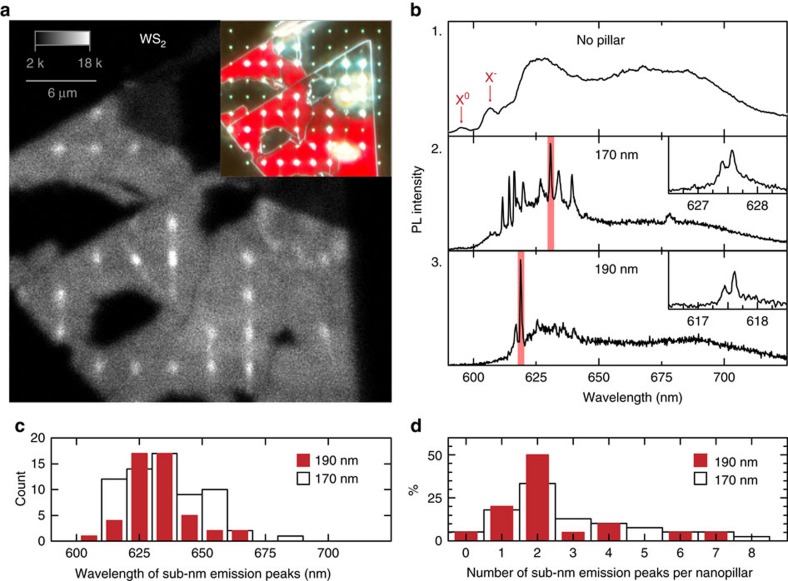
Creation of quantum-emitter arrays in 1L-WS_2_. (**a**) Integrated PL intensity raster scan of a 1L-WS_2_ flake deposited on top of a 3 μm spaced, 170-nm-high nanopillar array, taken at 300 nW μm^−2^, 532 nm laser excitation at 10 K. Colour-scale bar maximum is 18 kcounts s^−1^. Inset: true-colour DFM image of the same area. The red region corresponds to the WS_2_ monolayer. (**b**) PL spectra of 1L-WS_2_ at 10 K. Panel 1 shows a spectrum taken from a flat region away from nanopillars. Red arrows indicate unbound monolayer neutral (X^0^) and charged (X^−^) excitons. Panels 2 and 3 show representative spectra of WS_2_ on 170 and 190 nm nanopillars, respectively. Insets are high-resolution PL spectra of the red-highlighted spectral regions, showing the fine-structure splitting of the peaks. (**c**) Distribution of the emission wavelengths measured for 1L-WS_2_ QEs on 170 (black and white) and 190 nm (red) nanopillars. (**d**) Distribution of the number of narrow emission lines observed per nanopillar for 1L-WS_2_ QEs on 170 (black and white) and 190 nm (red) nanopillars. DFM, dark-field optical microscopy.

The method we present for QE creation is not restricted to a specific LM. We predict a similar effect on different LMs and test this by using 1L-WS_2_. Despite previous efforts to measure QEs in 1L-WS_2_, there has only been one previous report of single-photon emission in this material^22^. Figure 3a shows an integrated PL intensity raster scan map taken at ∼10 K of a 1L-WS_2_ on a substrate with 170-nm-high nanopillars square array spaced by 3 μm. The inset shows a true-colour dark-field optical microscopy image of the same flake, where the red areas (due to fluorescence) are 1L-WS_2_. Once again, the brighter spots correspond to the unpierced nanopillar locations, as verified by AFM measurements, and show overlap with the bright fluorescence spots in the PL intensity image where, similar to WSe_2_, intensity is increased (here by a factor ∼4) at every one of the 22 non-pierced nanopillar sites in the flake. Panel 1 of Fig. 3b shows the typical 1L-WS_2_ emission spectrum at ∼10 K (ref. 22), measured from a flat region of the same flake away from the nanopillars. The X^0^ and X^−^ unbound excitons are labelled in the figure, while the broad red-shifted emission band arises from weakly localized or defect-related excitons in the 1L-WS_2_ at low temperatures^22^, and is present in this material regardless of location. Panels 2 and 3 of Fig. 3b show representative PL emission spectra taken at nanopillars of heights ∼170 and∼190 nm, respectively, where once again sub-nm spectral features arise. We note that we observe fine-structure splitting for WS_2_ in these QEs, which range from 300 to 810 μeV (Supplementary Fig. 6), as represented in the panel insets corresponding to the spectral regions highlighted in red. We also measure the spectrum of several WS_2_ QEs as a function of time (see Supplementary Fig. 6) and find all spectral wandering values below 0.5 meV over 1–2 min. Figure 3c shows statistics on QE emission wavelength collected for over ∼80 QEs for 1L-WS_2_ on 170-nm (white bars) and 190-nm (red bars) nanopillars. The wavelength distribution of the sub-nm emission lines, typically in the 610–680 nm region (53–300 meV redshift from X^0^)^22^, is as narrow as ∼20 nm for the 190-nm nanopillars. Most nanopillar sites on WS_2_ show multiple sub-nm lines, suggesting the creation of several QEs at each site for these nanopillar heights. Figure 3d plots a histogram of the number of sub-nm peaks appearing at each nanopillar for both nanopillar heights. The trend is similar to that seen in WSe_2_, where higher nanopillars lead to a narrower spread in the number of peaks towards a higher likelihood of creating a single QE at each nanopillar site. We note that we obtain a 95% yield of QE creation in 1L-WS_2_ on non-pierced nanopillars. Further, ∼75% of these display two or less sub-nm emission peaks. In contrast, the 60- and the 130-nm-high nanopillars do not result in any QE occurrence (see Supplementary Fig. 7 for examples of these measurements).

## Discussion

We presented a simple method for the deterministic creation of scalable arrays of quantum-light emitters embedded in LMs emitting at different regions of the optical spectrum^33^. The reliability of the technique will accelerate experimental studies of QEs in TMDs, which at present rely on their rather rare and random occurrence^11^,^12^,^13^,^14^,^15^. In the immediate future, a detailed study is necessary in order to achieve a better understanding of the specific role of nanopillar height and geometry in defining the characteristics of the quantum emission. We expect tunability of the optical emission by varying the shapes of the underlying nanostructures. In this respect, interesting possibilities to realise dynamical circuits using micro-electro-mechanical systems and piezoelectric tuning exist. Heterostructures may enable new routes towards tunnel-coupled quantum devices and the formation of QE molecules. Several approaches are being investigated for the production of wafer-scale samples^34^,^35^, which could lead to rapid optimization. While our approach is already compatible with standard silicon processing techniques, it is nevertheless not restricted to the specific properties of the substrate. In fact, even nanodiamonds of the appropriate dimensions, drop-cast onto silica substrates, are able to create QEs in 1L-WSe_2_ (see Supplementary Fig. 8 and Supplementary Note 2). The choice of substrate material will be particularly important, for example, when considering inhomogeneous line-broadening due to charge noise. Further, the flexibility in the choice of substrate, in turn, provides an opportunity to create hybrid quantum devices where LM QEs can be coupled to quantum systems in other materials such as spins in diamond and silicon carbide.

## Methods

### Substrate preparation

The silica nanopillar substrate is fabricated with a high-resolution direct-write lithographic process via spin-on-glass polymer hydrogen silsesquioxane (HSQ)^36^. First, a wafer with 2 μm thermal oxide is cleaved and then cleaned. HSQ resist (FOx-16, Dow-Corning) is diluted with methyl isobutyl ketone (MIBK) in different ratios and spun onto the substrate, giving variable thickness depending on the dilution. After baking at 90 °C for 5 min, the substrate is exposed in an electron beam lithography tool (Elionix F-125) and then developed in a 25% solution of tetramethyl ammonium hydroxide (TMAH) developer and rinsed in methanol. To convert the defined structures into pure SiO_2_, we apply rapid thermal annealing at 1,000 °C in an oxygen atmosphere^37^, resulting in arrays of sharply-defined sub-100 nm silica nanopillars.

### Optical measurements

Room temperature Raman and PL measurements are carried out using a Horiba LabRam HR Evolution microspectrometer equipped with a × 100 objective (numerical aperture 0.9) and a spot size <1 μm. The pixel-to-pixel spectral resolution for the Raman measurements is ∼0.5 cm^−1^. Bragg gratings (BraggGrate) are used to detect the ultralow frequency Raman peaks. The power is kept below 50 μW to prevent heating effects. The excitation wavelength used is 514.5 nm for WSe_2_ and 457 nm for WS_2_.

A variable-temperature helium flow cryostat (Oxford Instruments Microstat HiRes2) is used to perform low-temperature PL measurements with a home-built confocal microscope mounted on a three-axis stage (Physik Instrumente M-405DG) with a 5-cm travel range, 200-nm resolution for coarse alignment and a piezo scanning mirror (Physik Instrumente S-334) for high-resolution raster scans. PL is collected using a 1.7-mm working distance objective with a numerical aperture of 0.7 (Nikon S Plan Fluor × 60) and detected on a fibre-coupled single-photon-counting module (PerkinElmer: SPCM-AQRH). Photon correlations from a Hanbury Brown and Twiss interferometer are recorded with a time-to-digital converter (quTAU). A double grating spectrometer (Princeton Instruments) is used for acquiring spectra. For PL measurements, the excitation laser (532 nm, Laser Quantum) is suppressed with a long pass filter (550 nm Thorlabs FEL0550).

### Data availability

The data that supports the findings of this study are available from the corresponding author upon request.

## Additional information

**How to cite this article:** Palacios-Berraquero, C. *et al*. Large-scale quantum-emitter arrays in atomically thin semiconductors. *Nat. Commun.* **8,** 15093 doi: 10.1038/ncomms15093 (2017).

**Publisher's note**: Springer Nature remains neutral with regard to jurisdictional claims in published maps and institutional affiliations.

## Supplementary Material

Supplementary Information Supplementary Figures, Supplementary Notes and Supplementary References

Peer Review File
