# Supplementary material for: Large-scale quantum-emitter arrays in atomically thin semiconductors
Source: Nat Commun. 2017 May 22;8:15093. doi: 10.1038/ncomms15093 (PMC5458119; doi:10.1038/ncomms15093)
Supplement: Supplementary Information — Supplementary Figures, Supplementary Notes and Supplementary References [file ncomms15093-s1.pdf]

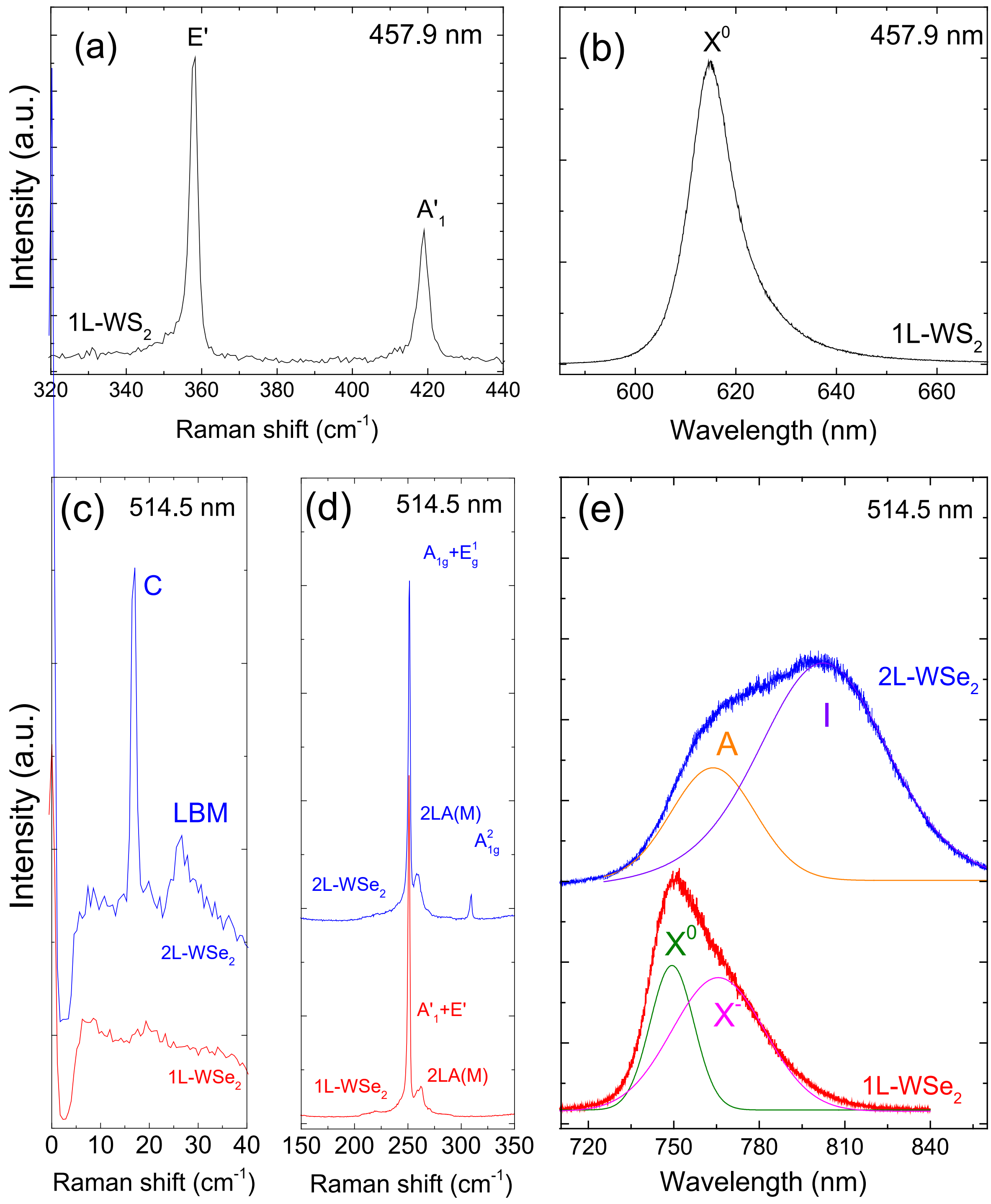

Supplementary Figure 1 | RT optical characterization of 1L-WS_2, 1L-WSe_2 and 2L-WSe_2. (a) Raman and (b) PL spectra of 1L-WS_2 (black lines) after transfer on the patterned substrates; (c), (d) Raman and (e) PL spectra of 1L-WSe_2 (red lines) after transfer on the patterned substrates and of a reference sample of 2L-WSe_2 on Si/SiO_2 (blue line). The excitation wavelength is 514.5 nm for WSe_2 and 457 nm for WS_2.

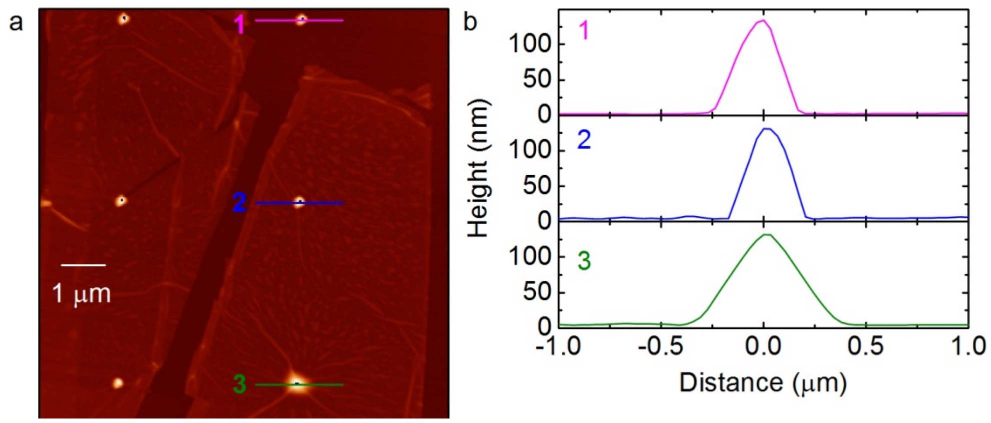

Supplementary Figure 2 | AFM characterisation of nanopillar sites. a, AFM scan of a 1L-WSe_2 on 130-nm nanopillars. Colour scale maximum is 135 nm. Nanopillars are labelled: 1 (outside the flake, bare), 2 (under the flake, pierced) and 3 (under the flake, non-pierced). **b,** Height profiles across the lines over nanopillars 1 (pink), 2 (blue) and 3 (green). The full-width half-maximum measured for the nanopillars with no flake on top (1 and 2) are ~250 nm, while that of site 3 is ~500 nm, larger by as much as a factor ×2 due to the tenting of the flake over the nanopillar. We correlate the dark field microscopy (DFM) images with AFM scans. As mentioned in the main text, non-pierced pillars appear as brighter spots in DFM due to a larger scattering area, compared to the dimmer pierced sites.

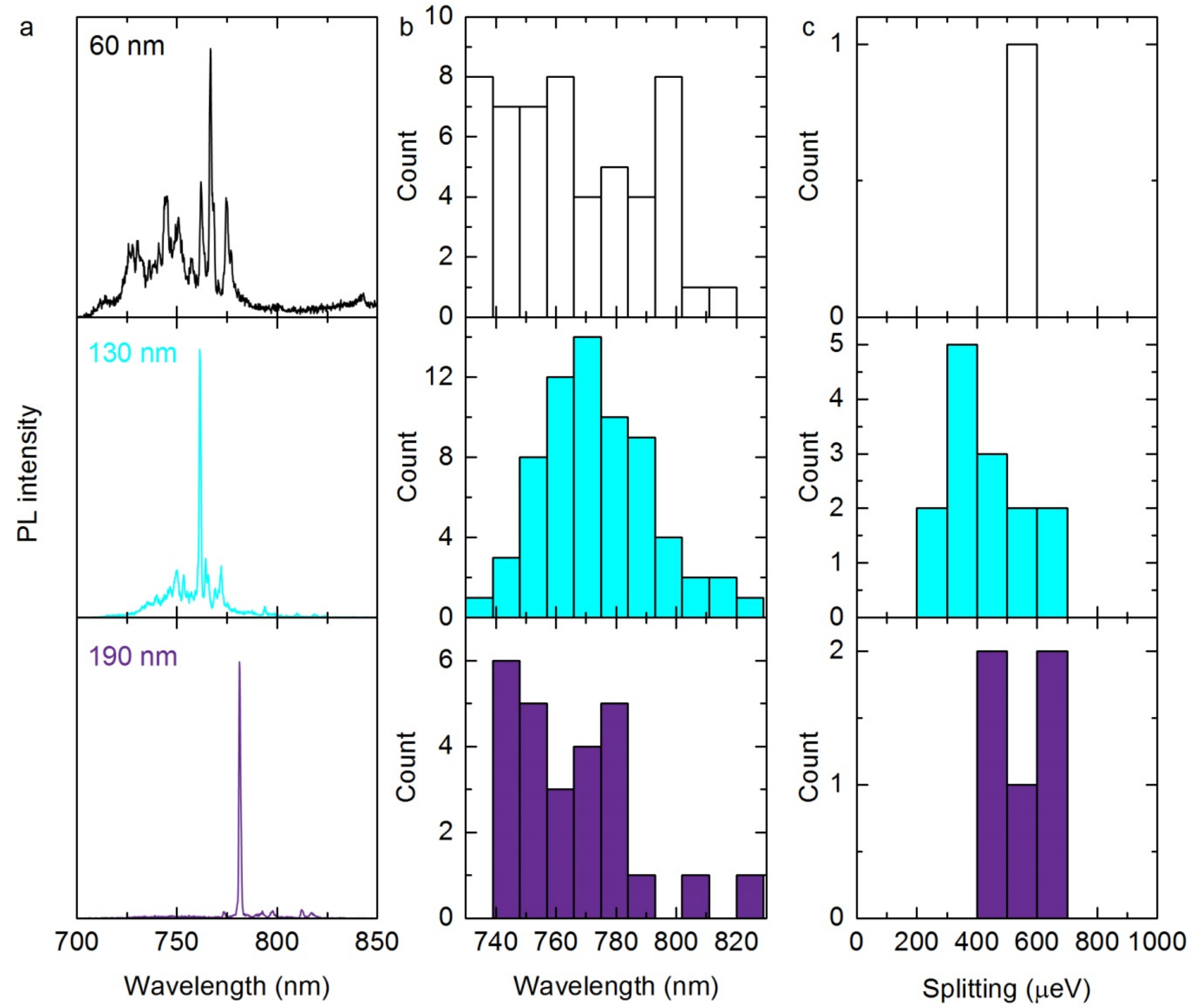

Supplementary Figure 3 | Effect of nanopillar height on 1L-WSe_2 QEs. Statistics for QEs measured on nanopillars of heights 60 nm (black), 130 nm (cyan) and 190 nm (purple). **a,** Example PL spectra for the different nanopillar heights taken at 10 K. As mentioned in the main text, the number of sub-nm peaks decreases for increasing height. **b,** Histograms showing emission wavelength of all sub-nm lines studied, which lie within the range of 730-820 nm. We carried out measurements on a total of over 80 nanopillar sites for the different nanopillar heights, observing no clear dependence of emission wavelength on nanopillar height, except a trend towards a narrower distribution of emission wavelength with increasing height. **c,** Fine structure splitting values of the 1L-WSe_2 QEs, which lie within the range 200-700 μeV. We measured one data point for the 60 nm nanopillars due to the QEs having linewidths in the range of 1 meV, generally greater than the fine structure splitting.

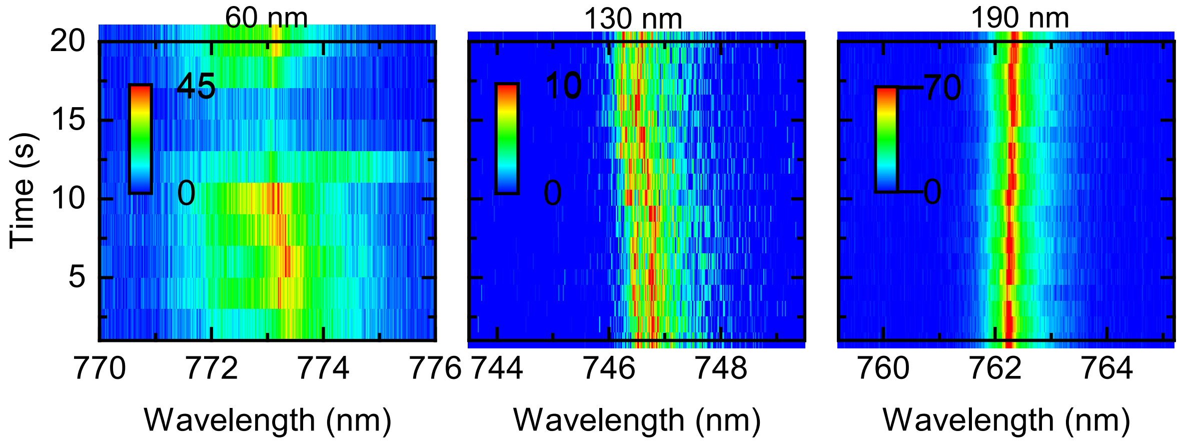

Supplementary Figure 4 | Spectral wandering of 1L-WSe_2 QEs as a function of nanopillar height. Spectra taken at 10 K of QEs on different nanopillar heights, measured using a high-resolution spectrometer grating (1800 gr/mm) over 20 s and showing a spectral window of 8 nm in each panel. The time resolution for each panel is 2, 1 and 1 s, respectively. Colour scale maxima are 45, 10 and 70 cts/s, respectively. Spectral wandering is reduced from ~1 to ~0.1 meV when going from 60 to 190-nm nanopillars. From this spectra, we extract the spectral wandering values in Figure 2e of the main text showing that QE spectral wandering decreases as the nanopillar height is increased.

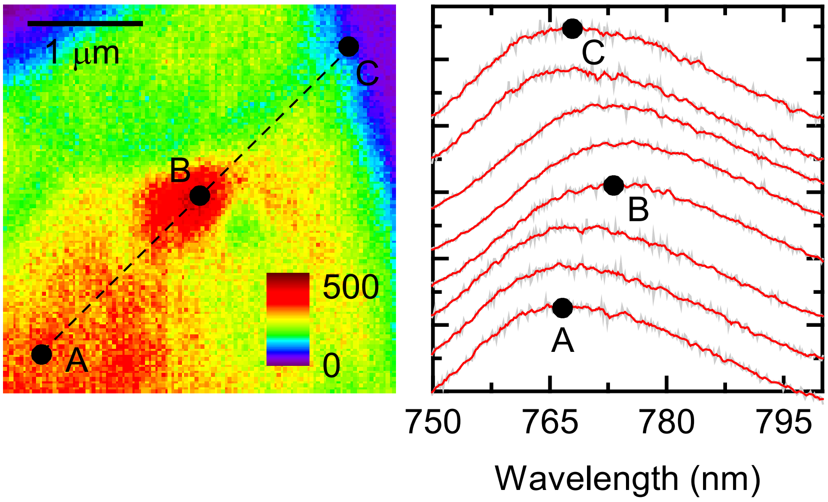

Supplementary Figure 5 | Room temperature 1L-WSe$_2$ nanopillar PL measurements. a, Integrated PL raster scan of integrated APD counts over one 130 nm-nanopillar site at RT, showing an increase in PL intensity at the nanopillar site (marked as B). Colour scale maximum is 590 kcounts/sec. The dashed line indicates where the spectra shown in the right panel are taken, with the first (A), central (B) and final (C) positions marked. **b,** RT spectra taken at the different positions along the dashed line in a, showing an X^0 redshift of ~8 nm (15 meV). In other samples we measured redshifts from the X^0 peak ranging from 3 to 15 nm, with no clear nanopillar height dependence.

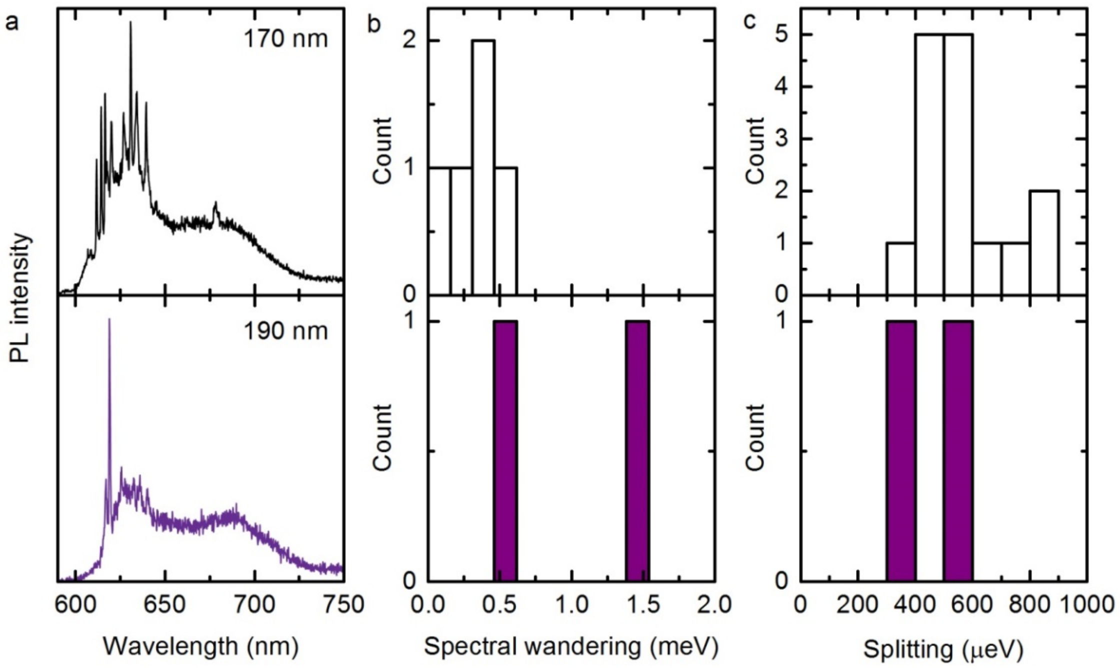

Supplementary Figure 6 | 1L-WS_2 QE statistics and effect of nanopillar height. We carried out PL experiments on WS_2 using 60, 130, 170 and 190 nm nanopillars, as shown in Figures 3c and 3d of the main text. We find QEs for 170 and 190-nm nanopillars. Data is shown in black for 170-nm nanopillars, and in purple for 190-nm nanopillars, at 10K. **a,** Representative spectra taken at each nanopillar height, showing a reduction in the number of sub-nm lines with nanopillar height. **b,** Spectral wandering as a function of nanopillar height, showing low values below 0.5 meV for 170 nm nanopillars, but no clear trend as a function of height. **c**, Fine structure splitting, previously unreported for WS_2 QEs, and lying in the same range as those values observed for WSe_2 QEs. The values are in the range 200 – 900 μeV, overlapping those observed in WSe_2 QEs.

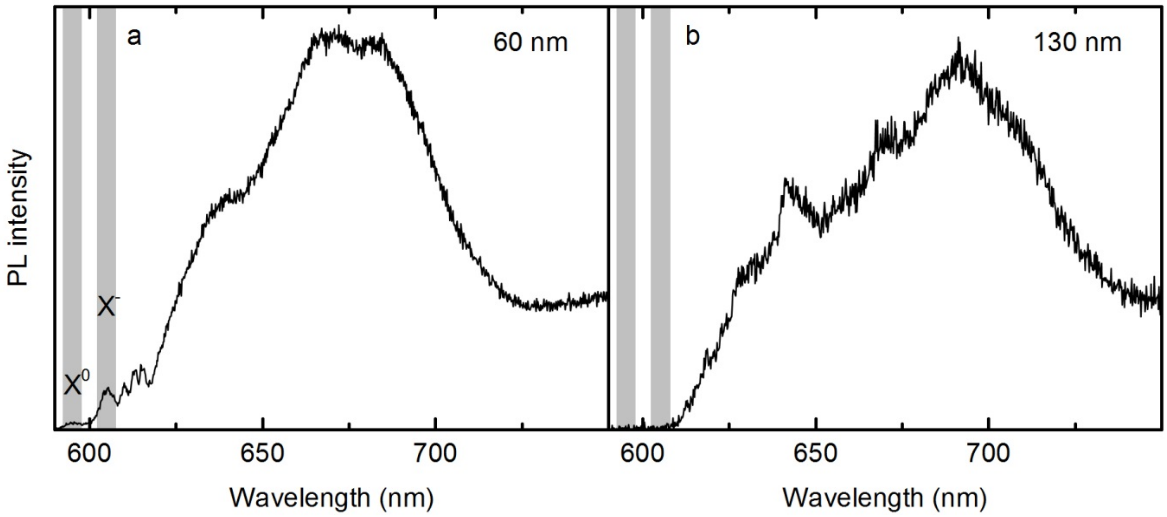

Supplementary Figure 7 | 1L-WS_2 on short nanopillars creating no quantum confinement. Spectra taken at nanopillar sites on 1L-WS_2, at 10K and using the same (532 nm) excitation as for all other PL measurements, on **a,** 60-nm and **b,** 130-nm nanopillars. No sub-nm peaks are observed. The X^0 and X^- bands are marked in grey for comparison. The broad red-shifted background seen in these scans is a feature seen in 1L-WS_2 at low temperature, due to weakly localised emission bands[7], in regions both over nanopillars and in flats areas.

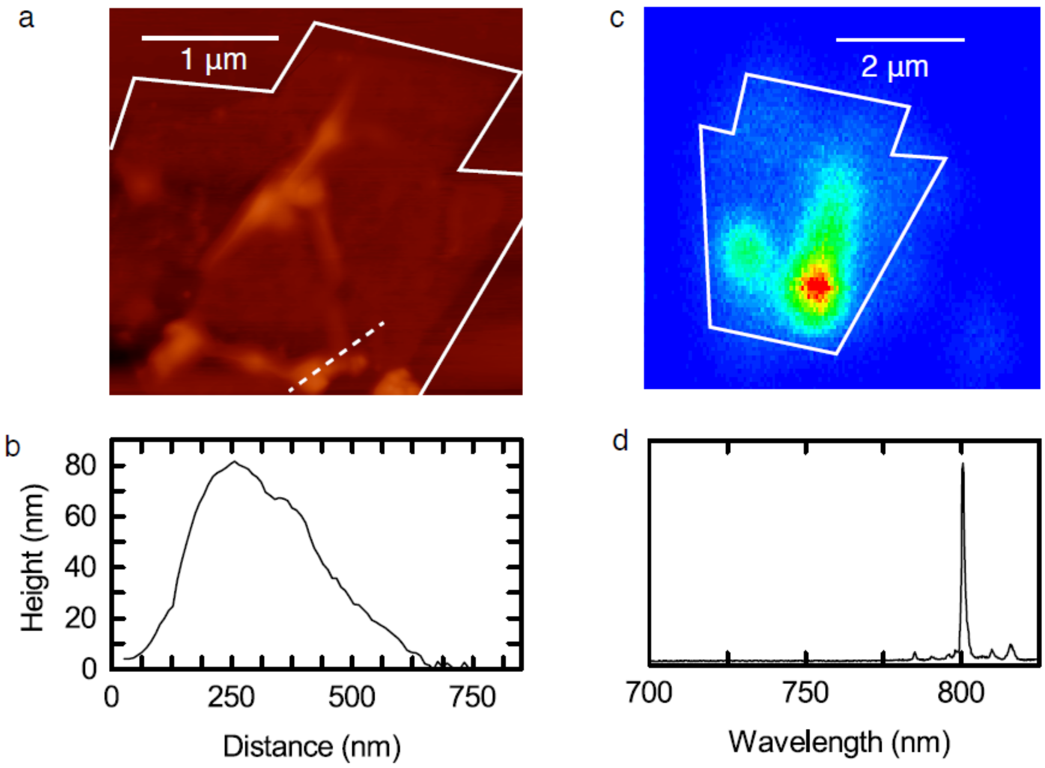

Supplementary Figure 8 | Nanodiamond substrates for QE creation. a, AFM scan showing 1L-WSe_2 on a silica substrate with drop-cast nanodiamonds. The elevated regions of the flake are caused by nanodiamonds below. **b,** Height profile at the nanodiamond site taken along the white dashed line in a. **c,** Raster scan of integrated PL at 10 K of the same 1L-WSe_2 as shown in a. Colour scale maximum is 43 kcounts/s, and corresponds to the nanodiamond site in b. **d,** PL emission at 10K from the same nanodiamond region as shown in a and c.

Supplementary Note 1. Raman and PL material characterisation

Room temperature Raman and PL measurements are performed as discussed in the main text.

Supplementary Figures 1a,b plot, respectively, the Raman and PL spectra of 1L-WS_2, as preliminarily identified by optical contrast, after transfer on the nanopillars. The Raman peaks at ~358 and ~419 cm^{-1} correspond to the E' and A'_1 modes, respectively[1]. The separation between the two peaks is thickness-dependent[2], and increases with increasing number of layers[2]. Our value of ~61 cm^{-1} is consistent with one-layer[2]. To further confirm this, we analyze its PL spectrum (Supplementary Figure 1b). A single peak at ~615 nm, corresponds to the neutral unbound exciton at the direct optical transition, a signature of 1L-WS_2[3]. We label this exciton X^0, following the notation used for TMDs by Ref. S4. We note that in literature one can find an alternative notation (e.g. in Ref. S3), where the letter A is used to distinguish it from a higher energy direct optical transition at ~520 nm (called "B") due to the spin-split valence band top.

Supplementary Figures 1c,d (red lines) plot the Raman spectrum of 1L-WSe_2, as initially identified by optical contrast, after transfer on the nanopillars. For comparison we also measure in Supplementary Figures 1c,d (blue lines) the spectrum of a 2L-WSe_2 flake on Si+285nm SiO_2, as identified by optical contrast. Supplementary Figure 1c indicates that in the low frequency Raman region two additional peaks appear at ~17 and ~26 cm^{-1} in 2L-WSe_2. The first peak, called C, is a shear mode caused by the relative motion of the layers, while the second peak is due to layer breathing modes[5,6] and can only appear in multi-layers. In Supplementary Figure 1d, red line, the peak at ~251 cm^{-1}, with full-width at half maximum (FWHM) ~2 cm^{-1}, is assigned to the convoluted A'_1+E' modes[1,2], degenerate in 1L-WSe_2[1,2], while the peak at ~262 cm^{-1} belongs to the 2LA(M) mode. Due to the A'_1 and E' degeneracy, we do not use the separation between peak positions as fingerprint of the number of layers. In 2L-WSe_2 (Supplementary Figure 1d, blue line), the A_{1g} and E_g^1 modes are degenerate at the same position of the peak in 1L-WSe_2, ~251 cm^{-1}, and the peak has the same FWHM, ~2 cm^{-1}. The position of the 2LA(M) mode instead blue shifts to ~ 259 cm^{-1}, consistent with an increasing number of layers[2]. We also note the appearance of a peak at ~309 cm^{-1}, corresponding to the A_{1g}^2 mode that emerges only in multilayer WSe_2[1,2]. In order to further confirm the number of layers, we analyse the PL spectrum of 1L-WSe_2 (Supplementary Figure 1e, red line). We identify two features, one at ~750 nm (Supplementary Figure 1e, green line), corresponding to the neutral unbound exciton X^0 of 1L-WSe_2[3,4] and a second at ~770 nm

(Supplementary Figure 1e, pink line), corresponding to the negatively charged unbound exciton X^- of 1L-WSe_2[4], as validated by its redshift of ~20 nm (the positively charged exciton would redshift ~10 nm from X^0 due to a smaller binding energy[4]). For reference, we compare the PL spectrum of 1L-WSe_2 to that of 2L-WSe_2 (Supplementary Figure 1e, blue line). The latter shows two components, at ~760 nm (orange line) and ~800 nm (purple line). The first corresponds to the direct optical transition, *A*[3], of 2L-WSe_2 , while the second is due to its indirect optical transition, *I*[3].

Supplementary Note 2. QE creation in 1L-WSe_2 using nanodiamonds

We deposit nanodiamonds, milled from bulk HPHT diamond (NaBond), of average diameter ~100 nm onto SiO_2/Si substrates. We do this via a standard drop-casting technique, whereby we suspend the nanodiamonds in ethanol and deposit a drop onto the substrate using a pipette. The drop is left on the substrate for 1 minute and then washed with de-ionised water, leaving behind only those nanodiamonds stuck to the surface of the substrate. We then place 1L-WSe_2 flakes on them using the same viscoelastic technique as reported in the main text. These create similar protrusions or deformations in the flake as the nanopillars, but of varying sizes owing to the size and shape dispersion of the nanodiamonds. Supplementary Figure 8a is an AFM scan of a 1L-WSe_2 flake on nanodiamonds. We take a height profile (shown in Supplementary Figure 8b) across the dashed line, where a nanodiamond is present under the flake. Supplementary Figure 8c shows an integrated PL raster scan of the same sample taken at 10 K. The flake is highlighted by the white lines. There is an increase in PL intensity at the nanodiamonds site, similar to the effect seen with the nanopillars. Supplementary Figure 8d shows a spectrum taken at this location, at 10 K and under 532 nm laser excitation, showing a sub-nm peak. About 20 such nanodiamonds-induced QEs were measured.

Supplementary References

1. Terrones, H. *et al.* New first order Raman-active modes in few layered transition metal dichalcogenides. *Sci. Rep.* **4,** 4215 (2014).
2. Zhao, W. *et al.* Lattice dynamics in mono- and few-layer sheets of WS2 and WSe2. *Nanoscale* **5,** 9677–83 (2013).
3. Zhou, B. *et al.* Evolution of electronic structure in Atomically Thin Sheets of WS2 and WSe2. *ACS Nano* **7,** 791–797 (2013).
4. Jones, A. M. *et al.* Optical generation of excitonic valley coherence in monolayer WSe2. *Nat. Nanotechnol.* **8,** 634–638 (2013).
5. Zhang, X. *et al.* Raman spectroscopy of shear and layer breathing modes in multilayer MoS 2. *Phys. Rev. B* **87,** 115413 (2013).
6. Wu, J.-B. *et al.* Interface Coupling in Twisted Multilayer Graphene by Resonant Raman Spectroscopy of Layer Breathing Modes. *ACS Nano* **9,** 7440–7449 (2015).
7. Palacios-Berraquero, C. et al. Atomically thin quantum light-emitting diodes. *Nat. Commun.* 7, 12978 doi: 10.1038/ncomms12978 (2016).
