# Supplementary material for: Large-scale quantum-emitter arrays in atomically thin semiconductors
Source: Nat Commun. 2017 May 22;8:15093. doi: 10.1038/ncomms15093 (PMC5458119; doi:10.1038/ncomms15093)
Supplement: Peer Review File [file ncomms15093-s2.pdf]

Editorial Note: this manuscript has been previously reviewed at another journal that is not operating a transparent peer review scheme. This document only contains reviewer comments and rebuttal letters for versions considered at Nature Communications.
